# The Attraction of the Dung Beetle *Anoplotrupes stercorosus* (Coleoptera: Geotrupidae) to Volatiles from Vertebrate Cadavers

**DOI:** 10.3390/insects11080476

**Published:** 2020-07-27

**Authors:** Sandra Weithmann, Christian von Hoermann, Thomas Schmitt, Sandra Steiger, Manfred Ayasse

**Affiliations:** 1 Institute of Evolutionary Ecology and Conservation Genomics, University of Ulm, 89069 Ulm, Germany; manfred.ayasse@uni-ulm.de; 2 Chair of Wildlife Ecology and Management, University of Freiburg, 79106 Freiburg, Germany; Christian.vonHoerman@npv-bw.bayern.de; 3 Department of Visitor Management and National Park Monitoring, Bavarian Forest National Park, 94481 Grafenau, Germany; 4 Department of Animal Ecology and Tropical Biology, Biocenter, University of Würzburg, 97074 Würzburg, Germany; thomas.schmitt@uni-wuerzburg.de; 5 Department of Evolutionary Animal Ecology, University of Bayreuth, 95447 Bayreuth, Germany; sandra.steiger@uni-bayreuth.de

**Keywords:** carrion decomposition, piglet cadaver, volatile organic compounds (VOCs), insect attraction, GC-EAD, synthetic cadaver volatiles

## Abstract

During decomposition, vertebrate carrion emits volatile organic compounds to which insects and other scavengers are attracted. We have previously found that the dung beetle, *Anoplotrupes stercorosus*, is the most common dung beetle found on vertebrate cadavers. Our aim in this study was to identify volatile key compounds emitted from carrion and used by *A. stercorosus* to locate this nutritive resource. By collecting cadaveric volatiles and performing electroantennographic detection, we tested which compounds *A. stercorosus* perceived in the post-bloating decomposition stage. Receptors in the antennae of *A. stercorosus* responded to 24 volatiles in odor bouquets from post-bloating decay. Subsequently, we produced a synthetic cadaver odor bouquet consisting of six compounds (benzaldehyde, DMTS, 3-octanone, 6-methyl-5-hepten-2-ol, nonanal, dodecane) perceived by the beetles and used various blends to attract *A. stercorosus* in German forests. In field assays, these beetles were attracted to a blend of DMTS, 3-octanone, and benzaldehyde. Generalist feeding behavior might lead to the super-dominant occurrence of *A. stercorosus* in temperate European forests and have a potentially large impact on the exploitation and rapid turnover of temporally limited resources such as vertebrate cadavers.

## 1. Introduction

In terrestrial ecosystems, vertebrate carrion and feces form unevenly distributed, ephemeral resource islands that are enriched with nitrogen, phosphorus, sulfur, and other vital elements, in contrast to the relatively nutrient-poor surroundings consisting of plant biomass [1,2]. These properties of dung and carrion, therefore, make them high-quality resources and hotspots of biological and chemical activity that microbes, insects, and other scavengers can utilize as their diet and for reproduction [2,3].

The decomposition of cadaver tissue, as the most dynamic and complex ephemeral resource patch, results in the release of volatile organic compounds (VOCs) arising from microbial putrefactive and decaying processes [4,5,6,7]. These VOCs are typically carboxylic acids (e.g., butanoic acid) and nitrogen-rich (e.g., skatole, indole) and sulfur-rich (e.g., dimethyl disulfide, dimethyl trisulfide (DMTS)) volatiles that play an important role in the attraction of necrophilous insects [4,7,8,9,10,11]. Each distinct decomposition stage can be characterized by specific carrion odor bouquets [10]. Since the intensity and the qualitative and quantitative composition of carrion scent varies over the whole decomposition period peaking in the most odoriferous phase at post-bloating stage [12], various insect groups are assumed to be successively lured toward characteristic odor profiles at specific decomposition stages. A plethora of studies have shown the colonization patterns of key insect groups on vertebrate carrion, mainly being published in journals of forensic entomology for post-mortem interval estimations [13,14,15,16,17]. House flies (Diptera: Muscidae), blow flies (Diptera: Calliphoridae), and flesh flies (Diptera: Sarcophagidae) are the first insects that visit a fresh cadaver and oviposit into its moist flesh to enable later hatching and feeding of larval masses. Later, when fly eggs and larvae are present in bloated and post-bloating stages, predators such as rove beetles (Coleoptera: Staphylinidae) appear at the cadaver to consume fly eggs and larvae. Following the bloated stage, the post-bloating stage is associated with the strongest olfactory signature [12]. This stage is also characterized by the opening of the body caused by the overpressure of microbial gases and of orifices formed by the feeding processes of insects and vertebrate scavengers. Consequently, the leakage of body fluids and the enhanced emission of VOCs increasingly attract further carrion-feeding insect taxa, such as burying beetles (Coleoptera: Silphidae) [10] and hide beetles (Coleoptera: Dermestidae) [18,19], until the advanced decay stage and even until the final stage of decomposition, when only dry material remains.

One group of insects that has gained evolutionary success by exploiting dung and carrion resources are dung beetles. In most cases, adult dung beetles feed and reproduce exclusively on feces. However, some dung beetle species shift from dung to alternative resources such as carrion (so called copronecrophagous feeding behavior), rotting fruits, fungi, and even living or dead millipedes [20,21,22,23,24]. Evidence has been presented that the shift from dung to carrion in large-sized neo-tropical dung beetles, mainly represented by the genera *Coprophanaeus*, *Deltochilum*, and *Canthon*, occurred recently and may have been caused by the extinction of mega-herbivores and consequently the disappearance of their dung droppings [21,25]. In South-East Asian tropical rain forests, mainly *Onthophagus* species shifted to vertebrate carrion [26], whereas in Africa, vertebrate carrion feeding in dung beetles was believed to occur rarely because of the competing presence of vultures and vertebrate scavengers such as hyenas and jackals. Indeed, Braack et al. [27] recorded 44 species of Scarabaeinae dung beetles that were attracted to an antelope cadaver. In European temperate regions, however, the niche of carrion utilization by dung beetles is mainly filled by large earth-boring dung beetles (Coleoptera: Geotrupidae). For example, *Anoplotrupes stercorosus*, *Geotrupes spiniger*, and *Trypocopris vernalis* have been found on adult pig cadavers [14] and *A. stercorosus* on rat carcasses [28].

*Anoplotrupes stercorosus* (Scriba, 1791) is a common occurring geotrupid beetle that can be found in European forests. This black beetle with its metallic blue coloration is relatively large (12–19 mm) and is active between June and September as a super-dominant species in thicket and in pole timber and mature stands [29,30,31,32]. *Anoplotrupes stercorosus*, described as a copronecrophagous species, mainly feeds on the fluid parts of animal excrement or carrion [33,34]. Several studies have shown the feeding preferences of *A. stercorosus* linked to herbivore dung. This species prefers sheep dung when allowed to choose among four herbivore dung types in laboratory and field experiments [35]. In a former study, the authors also observed that *A. stercorosus* locates cattle and horse dung [36]. These dung beetles are furthermore frequently found on human feces [33,37]. In contrast, only a few studies explicitly point out that geotrupid dung beetles are also capable of locating vertebrate cadavers as a resource at specific decomposition stages and are attracted in high numbers. Jarmusz and Bajerlein [38] showed, for the first time, that *A. stercorosus* and *T. vernalis*, as two common large European geotrupid beetles, locate pig carcasses in high numbers during the phase of strongest odor emission in various forest stands in Poland. However, carrion smell intensity was only evaluated via subjective olfaction by humans. Results from our previous study further support the occurrence of geotrupids dung beetles in high numbers at vertebrate carrion [34]. By collecting over 10,000 individual forest dung beetles (*A. stercorosus*) in pitfall traps baited with stillborn piglet cadavers in German forests, we showed that this beetle species is lured to carrion, and, more importantly, that it is mainly attracted to the progressed decomposition stages (Figures S1 and S2).

To our knowledge, no previous studies have revealed the means by which geotrupid dung beetles use volatiles to locate carrion as a resource. Thus, our aim was to characterize the compounds that occur in post-bloating cadaver odor bouquets and that can be perceived by the generalist dung beetle, *A. stercorosus*, by using gas chromatography coupled with electroantennographic detection (GC–EAD) techniques. Furthermore, we tested which of these EAD active compounds attract *A. stercorosus* by preparing various blends of synthetic cadaver volatiles out of six EAD active compounds and baiting pitfall traps to lure beetles in temperate forests in Germany. In addition, we recorded other insect species attracted to our artificial carrion VOC blends.

## 2. Materials and Methods

### 2.1. Piglet Cadaver Exposure and Dynamic Headspace Sampling of Cadaver Odor

Stillborn piglet cadavers (*Sus scrofa domestica*) were obtained from a local farmer near Ulm, Germany with permission being obtained through the “NecroPig” project within the framework of the Biodiversity Exploratories (http://www.biodiversity-exploratories.de [39]) and were subsequently frozen at −20 °C. In August 2015, we placed 12 piglet cadavers (1.4 kg average weight per piglet) in wire cages (63 cm × 48 cm × 54 cm, MH Handel GmbH, Munich, Germany) at six forest sites of the Exploratory Schwäbische Alb (South-West of Germany) for a total exposure time of one week. Data loggers (Thermochron iButton, Whitewater, WI, USA) that were placed inside of each cadaver cage recorded ambient air temperature every 30 min. Air temperature profiles of the surroundings of all cadavers were very similar and are shown in Figure S3. Decomposition stages were identified based on visual criteria [40]. We observed the following stages of terrestrial decomposition at all piglet cadavers: fresh, putrefaction, bloated, post-bloating, and advanced decay. Since the last decomposition stage, where only dry material remained, was not of interest for the current study, we did not determine this stage and terminated field work. Decomposition rate did not differ among cadavers, since we observed the same decomposition stages at each sampling day for each carrion.

Following carrion exposure, cadaveric odor bouquets were collected via dynamic headspace adsorption technique adapted after [41] at the post-bloating stage (day 6 post mortem (p.m.); Figure S4). To collect the headspace samples, we placed each piglet in a Toppits^®^ oven bag and closed it on both sides with a piece of wire. We connected the oven bag to a glass filter tube filled with an adsorbent material consisted of 10 mg Tenax-TA (mesh 60–80; Supelco, Bellefonte, PA, USA) and 10 mg Carbotrap B (mesh 20–40; Supelco). The adsorbents were fixed in the tubes using glass wool. The adsorbent filter was connected to a vacuum pump (DC12, Fürgut, Tannheim, Germany). The pump was turned on for a duration of 4 h with a flow rate of 200 mL/min, allowing incoming air to pass through the pre-cleaned adsorbent filters; see [11,19,42,43] for successful 4 h samplings. VOCs were thus trapped in the filter, kept in a cooler directly after collection and afterwards stored in the freezer at −40 °C. Volatiles trapped in the filters were eluted with 200 µL of a 9:1 mixture of pentane (Uvasol^®^, Merck, Darmstadt, Germany) and acetone (SupraSolv^®^, Merck, Darmstadt, Germany) into clean glass vials. We pooled all eluates from the sampling day in order to generate a representative cadaver odor bouquet for the post-bloating decomposition stage. The total volume of the eluted pool sample (~2.4 mL) equaled 12 piglets per 4 h of sampling per 200 µL solvent volume. We added 1 µg tridecane as an internal standard (stock solution: 100 μg/mL in pentane) to the eluted pool sample and kept it at −40 °C until chemical analysis. For a more in-depth description of the protocol used to obtain dynamic headspace sampling of cadaveric odor, see [11].

### 2.2. Gas Chromatography with Electroantennographic Detection (GC–EAD)

Gas chromatography coupled with electroantennographic detection (GC–EAD) was used to identify volatiles that were perceivable by receptors in the antennae of the forest dung beetle and that arose from cadaver odor bouquets at the post-bloating stage. The GC–EAD device consisted of a 7820A gas chromatograph (Agilent Technologies, Waldbronn, Germany) with a flame-ionization detector (FID) connected to an EAD setup (Syntech, Hilversum, The Netherlands). We used antennae of eight living dung beetles (*Anoplotrupes stercorosus*) that were caught near Darmstadt, Germany, were kept in plastic containers on humidified soil, and were fed weekly with horse dung. For successful antennal dissection and later preparation, limited beetle movement is imperative, and thus beetles were briefly kept at 4 °C to lower metabolism. Micro scissors and a razorblade were used to cut off one lamellate antenna at its base, and an additional incision was made at the tip of the antenna for later conductivity through the antenna. By clamping two small pieces of dental wax between the last three antennal segments, the antennal sensilla fields were exposed to the odor stimulus (stream loaded with volatiles) (Figure S5). Then, we mounted the whole antenna between two capillaries filled with insect Ringer solution (8.0 g NaCl + 0.4 g KCl + 0.4 g CaCl_2_ in 1000 mL demineralized water). We injected 1 µL of the eluted cadaver odor sample into the gas chromatograph equipped with a non-polar DB5-MS column (30 m length, 0.25 mm diameter, 0.25 µm film, Agilent Technologies, Waldbronn, Germany) by using hydrogen as a carrier gas (constant flow, 2.0 mL/min) at an initial temperature of 40 °C. After 1 min, the splitter was opened, and the oven temperature was increased by 7.5 °C/min to 300 °C (hold time: 46 min). While the cadaver odor sample was being exposed to the antennal receptors of the beetles, their antennal responses were simultaneously recorded with a GC–EAD program (Gc-Ead v. 1.2.5, Syntech, Hilversum, The Netherlands) at an EAD sensitivity of 0.5 mV. One antenna per beetle was used for GC–EAD recordings, and only reproducible peaks (significant responses with at least 5 repetitions) were marked as being EAD active. More information about electroantennographic detection can be obtained in [11].

### 2.3. Chemical Analyses (GC–MS)

The structural elucidations of electrophysiologically active compounds of the pooled headspace sample was based on gas chromatography/mass spectrometry (GC-MS) (7890 gas chromatograph coupled with 5975 mass spectrometer, Agilent Technologies, Waldbronn, Germany) with the same method as described for GC: a non-polar DB5-MS column (30 m length, 0.25 mm diameter, 0.25 µm film, Agilent Technologies, Waldbronn, Germany) using helium as a carrier gas (constant flow, 2.0 mL/min) at an initial temperature of 40 °C. After 1 min, the splitter was opened, and the oven temperature was increased by 7.5 °C/min to 300 °C (hold time: 46 min). The sample was analyzed by using Agilent ChemStation software (Agilent Technologies, Waldbronn, Germany). Chemical compounds were identified by comparisons of their mass spectra with the reference library from the NIST11 (NIST/EPA/NIH Mass Spectral Library 2011) and GC retention indices, which were calculated by using an n-alkane reference mixture and confirmed with published Kováts retention indices. 

### 2.4. Field Experiment—Attracting Dung Beetles with Synthetic Cadaver Mixtures

Our former study showed that *A. stercorosus* is mainly attracted to the progressed (post-bloating and advanced decay) stages of decomposing vertebrate cadavers [34]. Therefore, we aimed to attract *A. stercorosus* in field assays with a synthetic cadaver bouquet from a post-bloating decomposition stage. We selected six electrophysiologically active compounds known to occur frequently in the carrion odor of the post-bloating decomposition stage in vertebrate cadavers [11,44,45,46,47]: benzaldehyde, DMTS, 3-octanone, 6-methyl-5-hepten-2-ol, nonanal, and dodecane (see below for more details concerning the preparation of synthetic mixtures). Moreover, we considered not only major compounds emitted by piglet cadavers, since minor compounds can play a prominent role, as shown in other insect attraction systems [48]. Compounds that were probably derived from the forest environment (e.g., green leaf volatiles) were excluded.

**Figure 1:**
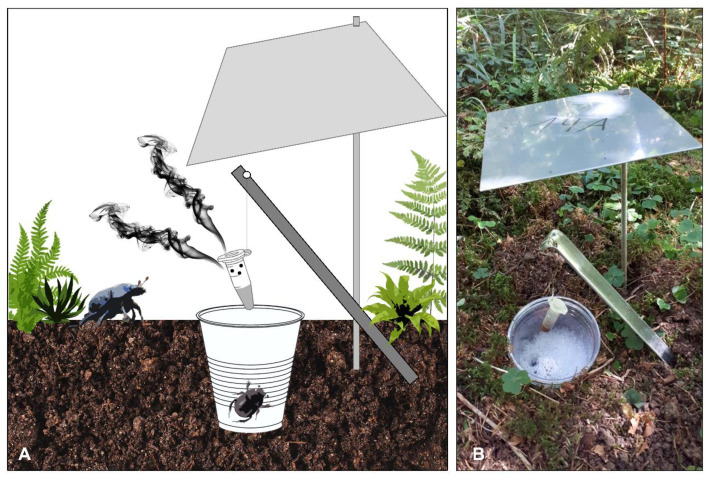
(**A**) Graphic illustration of a pitfall trap with bait (treatment) in a perforated Eppendorf tube used in the field assays and (**B**) the corresponding setup as an original image.

**Figure 2:**
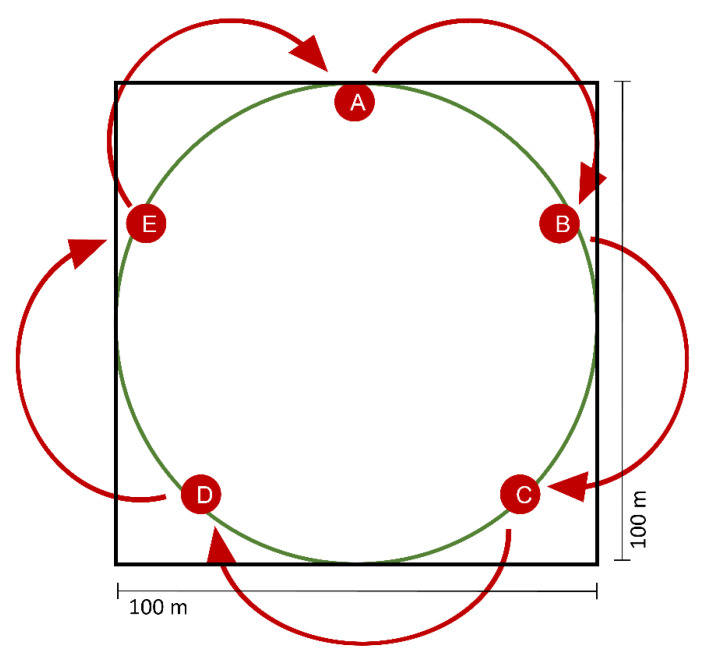
Location of five pitfall traps (A–E) on one forest plot (100 × 100 m). Traps were placed along the circumference of a circle with a maximal distance between each trap. For each baiting event, one specific treatment was positioned on a trap site, and at the next event, the treatment was rotated clockwise to the next trap position (shown as arrows) to avoid location effects.

To test the attraction of the complete synthetic cadaver mixture or subsets of the mixture for the forest dung beetle *A. stercorosus*, we performed field experiments in August 2018 at five test locations of the Exploratory Schwäbische Alb, Germany (forest plots of the Biodiversity Exploratories project, 100 × 100 m each, Figure S6). We located five pitfall traps (A–E) on each of the five plots (see Figure 1 and Figure 2). On each plot, the traps were placed along the circumference of a circle with a diameter of 100 m to maximize the distance between each trap. At each trap, a 2 mL Eppendorf tube filled with substances corresponding to the following five treatments was exposed to attract beetles:

- Treatment 1: a complete mix of all six EAD active compounds (benzaldehyde, DMTS, 3-octanone, 6-methyl-5-hepten-2-ol, nonanal, and dodecane), later described as “complete mixture”
- Treatment 2: three volatile EAD active compounds (benzaldehyde, DMTS, and 3-octanone), later described as “blend 2”
- Treatment 3: three volatile EAD active compounds (6-methyl-5-hepten-2-ol, nonanal, and dodecane), later described as “blend 3”
- Treatment P: positive control (a piece of piglet cadaver tissue (~1 cm^3^) in the post-bloating stage, previously cut from a decaying piglet and immediately frozen at −20 °C)
- Treatment N: negative control (empty tube).

According to their retention indices, the complete mixture consisting of six compounds (treatment 1) was separated into two blends with each of the three compounds included (treatment 2 and treatment 3). Each tube was connected to a piece of wire on a sand hook and was placed above a pitfall trap filled with scent-free detergent/water solution that was covered with a small plastic rain shield (Figure 1). To ensure the continuous emission of cadaver odor, we perforated the tubes at the upper 1 cm under the lid. Four holes per tube were made by using metal pins. The synthetic cadaver mixtures and the controls were exposed to lure insects for a total of 48 h. This procedure was repeated for five baiting events (N = 25 in total for each treatment), and after each event, a treatment was moved to the next trap in clockwise rotation to avoid site effects (Figure 2). Additionally, we paid special attention ensuring that between each plot, treatments were arranged next to treatments deviating in treatment number to prevent cross-interactions (Figure S7). After each baiting event, we emptied the traps, counted all lured insects, and identified them to species or family level using [33].

### 2.5. Preparation of Synthetic Cadaver Mixtures

Benzaldehyde, DMTS, 3-octanone, 6-methyl-5-hepten-2-ol, nonanal, and dodecane were mixed according to the quantitative compound relation in the natural cadaver headspace sample. To take account of the various physiochemical properties and vapor pressures of the synthetic compounds, we first mixed all these compounds according to the results of the quantitative chemical analyses and filled a total amount of ~250 µL of the blend into a perforated Eppendorf tube. Afterwards, we collected a headspace sample of this blend by using the same setup as described above, except that we placed the tube in a hermetic glass flask instead of an oven bag, eluted the compounds out of the filter, added 1 µg tridecane as an internal standard (stock solution: 100 μg/mL in pentane), and analyzed the sample by using gas-chromatography. Subsequently, we compared the new with the former blend and adjusted the blend stepwise to achieve as closely as possible the natural emission rate from the decaying piglet (Table S1). The purity of the synthetic substances ranged from 98 to 99% (benzaldehyde and nonanal: Merck, Darmstadt, Germany; DMTS, 3-octanone, 6-methyl-5-hepten-2-ol, and dodecane: Sigma-Aldrich, Munich, Germany).

### 2.6. Statistical Analyses

All statistical analyses were performed in R v. 3.5.2 [49]. Response variables showed non-normal distributions (*p* < 0.001), as assessed by Shapiro–Wilk normality tests (package “stats” [49]). Therefore, we carried out non-parametric Kruskal–Wallis tests (package “stats”) to find differences in the invertebrate attraction among all treatment groups and baiting events and post-hoc pairwise tests for multiple comparisons of mean rank sums after Nemenyi to identify which treatment was most attractive to the invertebrates (package “PMCMR” [50]). Post-hoc test after Nemenyi corrects for multiple samplings.

## 3. Results

### 3.1. Electrophysiology and Chemical Analyses

**Figure 3:**
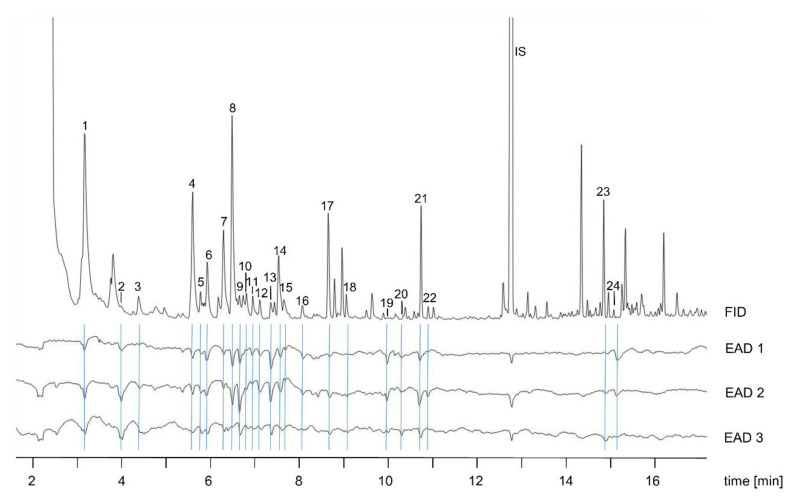
Electrophysiologically active compounds of a pooled headspace sample of piglet cadavers in post-bloating decay (6 days post mortem) by using antennae of *Anoplotrupes stercorosus*. Only reproducible peaks (significant responses on at least 5 repetitions) were marked as EAD active (consecutive numbering and blue lines). IS = internal standard, EAD = electroantennographic detection, FID = flame-ionization detector.

In the electrophysiological assessment of *A. stercorosus* antennae, we registered 24 EAD active compounds in the post-bloating decay headspace sample (Figure 3). In addition, we found the internal standard (tridecane) also to be electrophysiologically active.

We identified a total of 19 compounds using GC–MS analysis (Table 1). Dimethyl trisulfide (14.34%), methyl propyl disulfide (11.31%), and benzaldehyde (7.08%) were the dominant compounds in the post-bloating decay sample.

In the post-bloating decay odor bouquet, DMTS, 3-octanone, 1-methoxy-4-methylbenzene, camphor, and dodecene elicited the strongest antennal receptor responses. 

### 3.2. Field Experiment—Attracting Dung Beetles with Synthetic Cadaver Mixtures

**Figure 4:**
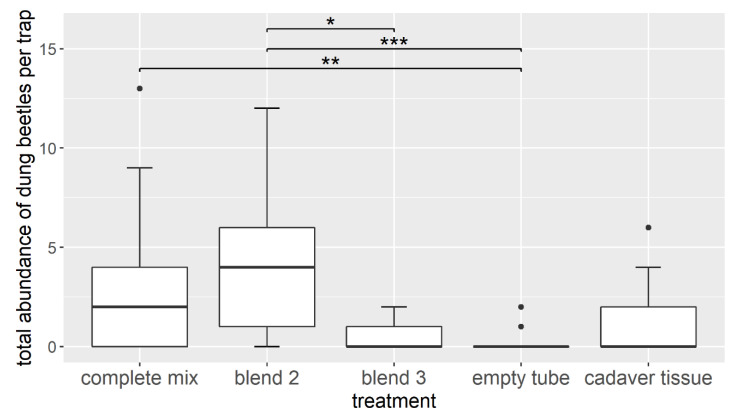
Comparison of the abundance of attracted *Anoplotrupes stercorosus* individuals among the different treatments (complete mix: all six EAD active compounds (benzaldehyde, dimethyl trisulfide, 3-octanone, 6-methyl-5-hepten-2-ol, nonanal, and dodecane), blend 2: three EAD active compounds (benzaldehyde, dimethyl trisulfide, and 3-octanone), blend 3: three EAD active compounds (6-methyl-5-hepten-2-ol, nonanal, and dodecane), empty tube: negative control, cadaver tissue: positive control). Each box shows the median, 75% percentile, 25% percentile, and highest and smallest non-extreme value within a category, and asterisks indicate significant differences between treatments (Kruskal-Wallis test: χ^2^ = 31.077, df = 4, *p* < 0.001; post-hoc Nemenyi tests (*p* < 0.05): blend 2 vs. blend 3: *p* = 0.002, complete mix vs. empty tube: *p* = 0.005, blend 2 vs. empty tube: *p* < 0.001; significance levels: ns (*p* > 0.05), * *p* ≤ 0.05, ** *p* ≤ 0.01, *** *p* ≤ 0.001).

In total, we lured 220 individuals of *A. stercorosus* in all pitfall traps and at all five baiting events combined. The treatments had a significant effect on the total beetle abundance per trap (Kruskal–Wallis test: χ^2^ = 31.077, df = 4, *p* < 0.001, Figure 4). The complete synthetic cadaver mixture (treatment 1 with 74 individuals) and blend 2 (treatment 2 with 94 individuals) both attracted more forest dung beetles compared with other treatments. Most attractive was blend 2 (treatment 2 vs. 3: *p* = 0.002, treatment 2 vs. negative control: *p* < 0.001); however, this did not significantly differ from the complete mixture, as the second most attractive bait. Both mixtures lured significantly more beetles than the negative control (complete mixture vs. negative control: *p* = 0.005, treatment 2 vs. negative control *p* < 0.001). Concerning attracted *A. stercorosus* specimens, we found the lowest attractiveness in blend 3 (treatment 3 with 14 individuals), the negative control (7 individuals), and the positive control (34 individuals).

In addition, we collected 5984 specimens of other insects and invertebrate groups from all treatments and all baiting events combined (burying beetles (Silphidae), rove beetles (Staphylinidae), flies (Scathophagidae, Calliphoridae, Muscidae, Sarcophagidae), slugs, ground beetles (Carabidae), spiders, wasps, ants, and isopods; for composition of certain treatments see Table S2). With regard to the total abundance over all groups, blend 2 (treatment 2, 3078 individuals) was the most attractive, followed by the complete synthetic cadaver mixture (treatment 1, 2399 individuals), the positive control (treatment P, 281 individuals), blend 3 (treatment 3, 251 individuals), and last, the negative control (treatment N, 195 individuals). Invertebrate taxa displayed contrasting dynamic responses to the treatments. Significant differences were found for the attraction of the following groups: *A. stercorosus*, Silphidae, Staphylinidae, flies (Scathophagidae, Calliphoridae, Muscidae, Sarcophagidae), and slugs (all *p* < 0.001, Table S3). However, all other groups (Carabidae, spiders, wasps, ants, and isopods) seemed to be accidentally attracted, as no significant difference was seen in the abundance for the different treatments (all *p* > 0.05). We found that, in all insect groups that showed a significant difference in responsiveness towards the baits, most individuals were attracted by blend 2, followed by the complete mixture (treatment 1). No significant effect was observed in the total catch rate per baiting event (Figure S8).

## 4. Discussion

Our results demonstrate that the antennae of the forest dung beetle *A. stercorosus* respond to 24 volatiles from post-bloating decay headspace samples of stillborn piglet carrion odor. The strongest responses were elicited from DMTS, 3-octanone, and dodecene. In tests of mixtures of six EAD active substances in the field, *A. stercorosus* was most attracted to the blend consisting of DMTS, 3-octanone, and benzaldehyde and slightly but non-significantly less attracted to the complete mixture (benzaldehyde, DMTS, 3-octanone, 6-methyl-5-hepten-2-ol, nonanal, and dodecane). Moreover, various other carrion-associated insect and invertebrate groups were also lured to this blend.

### 4.1. Perception of Volatile Carrion Odor Components

In our electrophysiological analyses, we examined VOCs from headspace samples of piglet cadavers during post-bloating decay. We identified 19 VOCs the forest dung beetle *A. stercorosus* is able to perceive. Dimethyl trisulfide, 3-octanone, and dodecene elicited the strongest responses in *A. stercorosus* antennae.

Dimethyl trisulfide (DMTS) is well known to play a role in insect attraction towards decomposing animal tissue [6]. For example, GC–EAD recordings revealed that DMTS allows the burying beetle *Nicrophorus vespilloides* to perceive a carcass at various decomposition stages [11,51]. Four blowfly species and, amongst them, gravid females of the blowfly *Lucilia sericata*, can verifiably perceive DMTS in electroantennographic detection assays and have been demonstrated to use this compound during experiments to locate rat carrion as suitable oviposition sites [52,53]. However, so far, no previous studies have been performed on the antennal responses of geotrupid dung beetles to sulfur-containing volatiles. Antennal responses have only been shown for the Japanese dung beetle *Geotrupes auratus* to five dung-specific volatiles without sulfur, namely 2-butanone, phenol, p-cresol, indole, and skatole [54]. DMTS is a metabolite of the microbial degradation of the sulfur-containing amino acids cysteine and methionine [55] and is a common cadaveric compound in vertebrate decay [47]. The compound 3-octanone also elicited high responses in our electrophysiological analyses. This compound, which smells like mushrooms (arising from various sources including fungi; [56]) has been described in dung volatiles of the New Zealand’s weka rail (*Gallirallus australis*) [57] and also in mouse carcass volatiles [58]. *Nicrophorus vespilloides*, a necrophagous beetle, can also perceive 3-octanone from dead piglet odor bouquets [11]. A further GC–EAD active compound, dodecene, has so far only been described in dung odor from white rhino [59] with a double bond at the third C-atom and in the scent of the clothing textiles from decomposing pigs used as training material for detection dogs to locate human remains [44]. To the best of our knowledge, this is the first study in which the antennal perception of dodecene has been described in carrion-associated insects, although the double bond position has still to be determined.

*Anoplotrupes stercorosus* beetles are highly attracted to progressed decomposition stages in field assays [34,38] and in our study, we have shown that the antennae of *A. stercorosus* respond to various compounds in the post-bloating headspace sample. Those compounds might explain the ability of *A. stercorosus* to discriminate between decomposition stages. Based on the high number of volatiles that *A. stercorosus* is able to perceive, this beetle species might use a blend of compounds instead of single volatiles for resource location and for the discrimination between decomposition stages. However, *A. stercorosus* beetles might also employ the higher concentration of a given volatile to discriminate between decomposition stages [38], as the concentration of DMTS, for example, increases over the course of vertebrate decomposition (see Figure 3 in [11]). In order to show the importance of blends versus single compounds, future studies should involve field assays with single VOCs and VOCs in mixtures.

Herbivore dung releases different VOCs from those in carrion [57]. Fifty-one common volatiles emitted from four herbivore dung types have been identified [60]; we have found six of these volatiles in our volatiles to be GC–EAD active, namely α-pinene, camphene, decane, limonene, nonanal, and dodecane. The overlap of some compounds of two types of ephemeral resources supports the assumption that carrion and dung have some odor volatiles in common that can be perceived by *A. stercorosus*. Nevertheless, the major herbivore dung VOCs are butyric acid, 2-butanone, skatole, indole [61], and cresol [60] and not the overlapping VOCs. Therefore, minor volatiles, which occur in feces and in carrion, might play a role in the perception of *A. stercorosus* when locating a resource. However, *A. stercorosus* might additionally perceive volatiles that are specific in dung odor bouquets.

In our study, the receptors in the antennae of *A. stercorosus* also responded to monoterpenes such as α-pinene and camphor, compounds that are well known to occur in plants [62,63]. Since we collected our headspace samples in natural ecosystems such as forests, these substances might have been released by vegetation in close vicinity to our cadavers. If *A. stercorosus* uses these volatiles as a background odor to orient within forest habitats has still to be investigated. This is, to the best of our knowledge, the first study in which the olfactory perception of a geotrupid beetle towards vertebrate carrion odor has been investigated via GC–EAD. Thus, species-specific studies are lacking until now.

### 4.2. Attractiveness of Selected Carrion Odor Compounds in Field Assays

In our field assays, forest dung beetles were most attracted to the blend consisting of benzaldehyde, DMTS, and 3-octanone, although all synthetic mixtures appeared to be enticing. This supports the hypothesis of choosy generalism, the selection of more valuable resources in the case of availability, in the feeding behavior in dung beetles, as shown in previous studies [35,36,64]. DMTS and 3-octanone also elicited the strongest electrophysiological responses in this beetle species, thus emphasizing that these two compounds probably play a prominent role in beetle attraction. DMTS, as a single compound, is sufficient to attract various carrion-associated blowfly species [53] and 3-octanone, as a characteristic fungal VOC, is known to be involved in the attraction of predatory beetles [65]. *Anoplotrupes stercorosus* beetles might also use 3-octanone as an olfactory cue to locate carrion, since fungal scent is a reliable indicator for the decomposition process of organic material [66]. Further field tests with single VOCs such as 3-octanone could clarify its role in attracting dung beetles. Benzaldehyde is derived from the metabolic degradation of amino acids, fatty acids, alcohols, and pyruvate [67] and is a common compound emitted by various organisms, e.g., it is also found in floral scents [62]. Benzaldehyde has been identified in the defensive secretions of millipedes, which function as an attractant of the Mexican carrion ball roller scarab *Canthon morsei* (Coleoptera: Scarabaeidae) [68]. Because benzaldehyde is extremely common and is therefore an unspecific volatile, we suggest that it plays a less important role in beetle attraction than DMTS and 3-octanone.

Remarkably, the complete mixture (treatment 1) and blend 2 were also most attractive to other cadaver-associated invertebrate groups [69] such as burying beetles (Silphidae), rove beetles (Staphylinidae), flesh, blow, and house flies (Scathophagidae, Calliphoridae, and Muscidae), dung flies (Sarcophagidae), and slugs. These findings highlight the attractive effect of the synthetic blends for carrion-associated invertebrates. Furthermore, blend 2 and, especially, DMTS and 3-octanone might resemble omnipresent cadaveric key compounds that are used by many carrion-related insect and invertebrate groups to locate this valuable resource. In contrast, ground beetles (Carabidae), spiders, wasps, ants, and isopods, which are commonly not strictly associated with cadavers, showed no preference for the synthetic volatile blends.

Compared with blend 2, blend 3, consisting of 6-methyl-5-hepten-2-ol, nonanal, and dodecane, was significantly less attractive to *A. stercorosus* beetles and other carrion-related invertebrates; the reason for this remains unclear. Nonanal has been found in sheep and horse dung and dodecane in cattle, horse, and boar dung [60] and in decaying mouse and pig carcasses [70,71]. Nonanal is further associated with detection by blow flies [70]. The role of nonanal as a semiochemical has been shown in many studies [72], but an attractive effect in dung beetles has never been published.

In our field assays, we found that carrion bait as a positive control lured, besides *A. stercorosus*, fewer insect specimens than all the synthetic mixtures that we tested, whereas in other studies natural baits such as fresh feces or carrion normally attract more insects than synthetic baits [64]. We assume that the decomposed tissue of 1 cm^3^ in size within each trap did not smell strongly enough for reproducible tests in the field, and thus larger tissue samples should be employed in future studies. However, with the aim to efficiently monitor whole dung beetle communities, a similar experimental setup providing dung instead of carrion as bait can be used.

## 5. Conclusions

Overall, our study showed that receptors in the antennae of the forest dung beetle *A. stercorosus* respond to various VOCs of post-bloating odor bouquets in electroantennographic tests. We have also found that the copronecrophagous beetle species *A. stercorosus* is attracted to the synthetic mixture of EAD active compounds DMTS, 3-octanone, and benzaldehyde in the field. DMTS and 3-octanone seem to be universal cadaveric compounds to which many cadaver-associated insects and other invertebrates respond. Therefore, we conclude from our study that only a few scent compounds out of a complex cadaveric odor bouquet, including DMTS and 3-octanone, are needed to lure *A. stercorosus* to decomposing resources such as carrion. *Anoplotrupes stercorosus* seems to be a super-opportunist and feeds on both resources, namely dung and carrion, explaining its copronecrophagous feeding behavior. If such ephemeral resources are scarce and unevenly distributed, generalist feeders as *A. stercorosus* can, in the long-term, be more successful than specialists that are dependent on one or the other substrate. Hence, this strongly opportunistic behavior might explain the super-dominant appearance of *A. stercorosus* on carrion in temperate European forest ecosystems and point towards a potentially great impact on the utilization of ephemeral resources such as vertebrate cadavers. In future studies, the dung VOCs that attract highly specialized dung beetles should be investigated and compared with those that we found to lure more generalist species such as *A. stercorosus*.

## Figures and Tables

**Table 1:** Relative amounts (in%) of all GC–EAD active compounds perceived by *Anoplotrupes stercorosus* in the pooled headspace sample of piglets in post-bloating decay (6 days post mortem).

No.	Compound Name	RI	Relative Amount (%) in Post-Bloating Decay (6 days p.m.)
1	unknown (artifact)	-	22.77
2	unknown	-	6.4
3	unknown	-	1.27
4	methyl propyl disulfide	927	11.31
5	α-pinene	931	0.83
6	camphene	948	5.23
7	benzaldehyde *	960	7.08
8	dimethyl trisulfide *	971	14.34
9	3-octanone *	984	1.88
10	6-methyl-5-hepten-2-ol *	991	1.89
11	decane	1000	1.92
12	3-carene	1008	1.4
13	1-methoxy-4-methylbenzene	1019	1.01
14	limonene	1027	1.4
15	benzyl alcohol	1032	5.29
16	butylbenzene	1055	0.93
17	methyl pentyl disulfide	1084	3.04
18	nonanal *	1104	1.23
19	camphor	1151	0.23
20	ethyl pentyl disulfide	1167	0.44
21	dodecene ^1^	1192	4.52
22	dodecane *	1200	0.64
	tridecane ^2^	1300	
23	unknown	1423	4.62
24	unknown	1437	0.34

* selected for field assay, RI = retention index, ^1^ unknown double bound position, ^2^ internal standard.

## Supplementary Materials

The following are available online at https://www.mdpi.com/2075-4450/11/8/476/s1, Figure S1: Forest dung beetles (*Anoplotrupes stercorosus*) on a piglet cadaver in a German forest (Schorfheide–Chorin region), Figure S2: Mass assemblages of *Anoplotrupes stercorosus* at two piglet cadavers in (a) post-bloating and (b) advanced decay stage (forests in Schorfheide–Chorin region). Beetles underwent feeding on cadaveric fluids and almost became immobilized in the wet soil, Figure S3: Temperature profile of the surroundings of 12 exposed piglet cadavers (01–12) over seven days. HS = Headspace sampling on day 6 after exposition. Decomposition stages: F = fresh, P = putrefaction, B = bloated, PB = post-bloating, AD = advanced decay, Figure S4: Sampling setup of dynamic headspace for cadaveric volatile organic compounds (VOCs) of a fresh piglet cadaver. In our experiment, we collected cadaver odor bouquets from piglets in post-bloating decay, Figure S5: A lamellate antenna of the dung beetle *Anoplotrupes stercorosus* was fixed between two capillaries of the electroantennographic device. Two small dental wax pieces kept the lamella open for the incoming stimulus (cadaveric odor stream) towards the receptors, Figure S6: Location of the five forest plots AEW (Alb Exploratory Wald (*engl*. Forest) 14, 33, 34, 46, and 48 in the Schwäbische Alb near Gomadingen (GPS: 48°23′57.524″ N 9°23′28.306″ E) and Münsingen (GPS: 48°24′41.205″ N 9°29′52.929″ E) where we conducted our field assays, Figure S7: Baiting procedure across space (left to right: all five forest plots AEW 14, 33, 34, 36, 38) and time (five baiting events 1–5). Treatments 1, 2, 3, N, and P were rotated clockwise after each baiting event to avoid location effects. We paid special attention to ensure that, between each plot, treatments were arranged next to treatments deviating in treatment number to prevent cross-interactions. Treatment description: treatment 1—complete mixture of all six EAD active compounds (benzaldehyde, dimethyl trisulfide, 3-octanone, 6-methyl-5-hepten-2-ol, nonanal, and dodecane), treatment 2—three EAD active compounds (benzaldehyde, dimethyl trisulfide, and 3-octanone), treatment 3—three EAD active compounds (6-methyl-5-hepten-2-ol, nonanal, and dodecane), treatment N—empty tube as negative control, treatment P—cadaver tissue in post-bloating stage as positive control, Figure S8: No significant difference of the total catch rate (total abundance of all lured invertebrates per trap) was observed among all baiting events (1–5) (Kruskal–Wallis test: χ^2^ = 6.098, df = 4, *p* = 0.192). Each box shows the median, 25% percentile, 75% percentile, and highest and smallest non-extreme value within a category, Table S1: GC–EAD active compounds from the headspace sample pool used for the field assays. In the table we show their original total amounts (µg), their calculated pipette scheme to a sum of 250 µL, and adjusted pipette scheme (µL), together with the proportional pipette schemes for treatments 1–3, Table S2: Abundance of insect and invertebrate taxonomic groups that were attracted by different treatments (1, 2, 3, N, and P), Table S3: Significant differences in various attracted insect and other invertebrate taxonomic groups among the different treatments (1, 2, 3, N, and P).
