# Supplementary material for: The Attraction of the Dung Beetle Anoplotrupes stercorosus (Coleoptera: Geotrupidae) to Volatiles from Vertebrate Cadavers
Source: Insects. 2020 Jul 27;11(8):476. doi: 10.3390/insects11080476 (PMC7469141; doi:10.3390/insects11080476)
Supplement: Supplementary file 1 [file insects-11-00476-s001.pdf]

Supplementary Materials

The attraction of the dung beetle *Anoplotrupes stercorosus* (Coleoptera: Geotrupidae) to volatiles from vertebrate cadavers

Sandra Weithmann [1,*], Christian von Hoermann [2,3], Thomas Schmitt [4], Sandra Steiger [5] and Manfred Ayasse [1]

[1] Institute of Evolutionary Ecology and Conservation Genomics, University of Ulm, 89069 Ulm, Germany; manfred.ayasse@uni-ulm.de (M.A.)

[2] Chair of Wildlife Ecology and Management, University of Freiburg, 79106 Freiburg, Germany;

[3] Department of Visitor Management and National Park Monitoring, Bavarian Forest National Park, 94481 Grafenau, Germany; Christian.vonHoerman@npv-bw.bayern.de (C.v.H.)

[4] Department of Animal Ecology and Tropical Biology, Biocenter, University of Würzburg, 97074 Würzburg, Germany; thomas.schmitt@uni-wuerzburg.de (T.S.)

5 Department of Evolutionary Animal Ecology, University of Bayreuth, 95447 Bayreuth, Germany; Sandra.Steiger@uni-bayreuth.de (S.S.)

* Correspondence: sandra.weithmann@uni-ulm.de (S.W)

The supplementary material is structured in sections Materials and methods and Results.

Materials and Methods

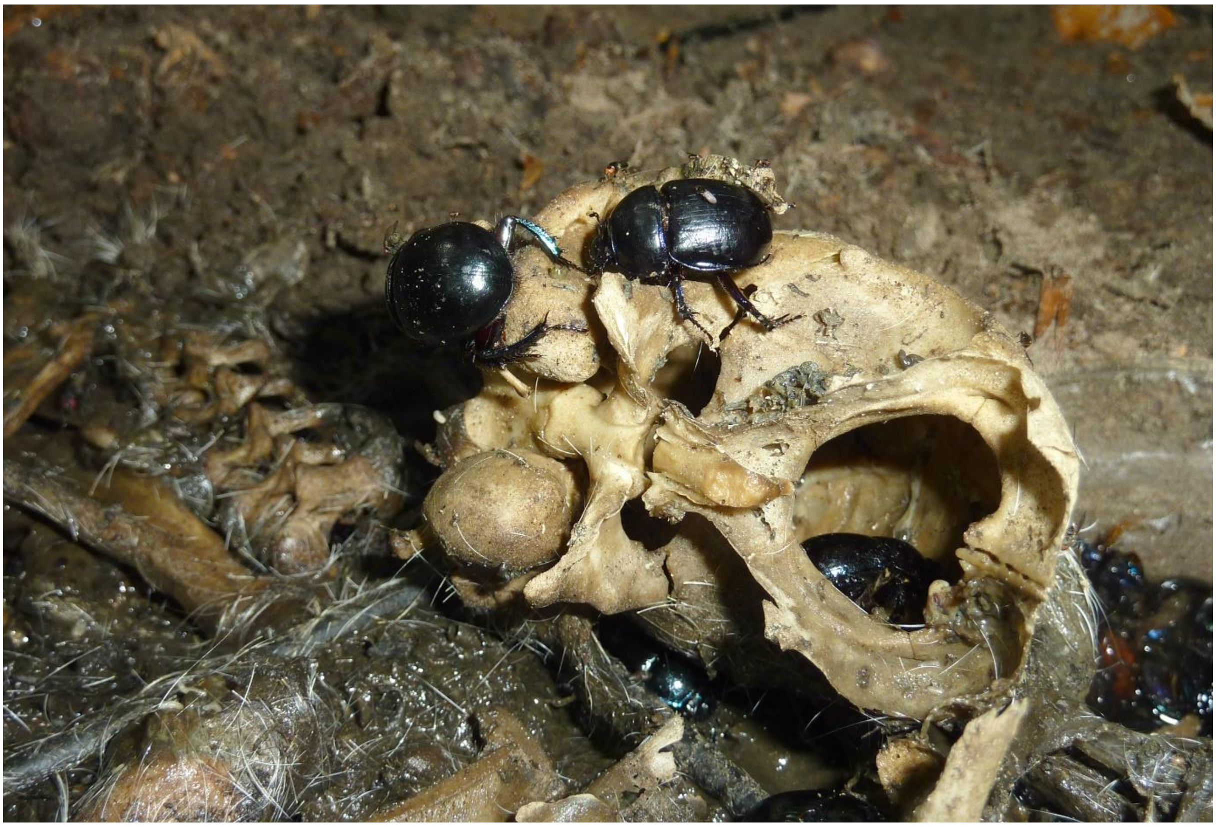

Figure S1. Forest dung beetles (*Anoplotrupes stercorosus*) on a piglet cadaver in a German forest (Schorfheide-Chorin region).

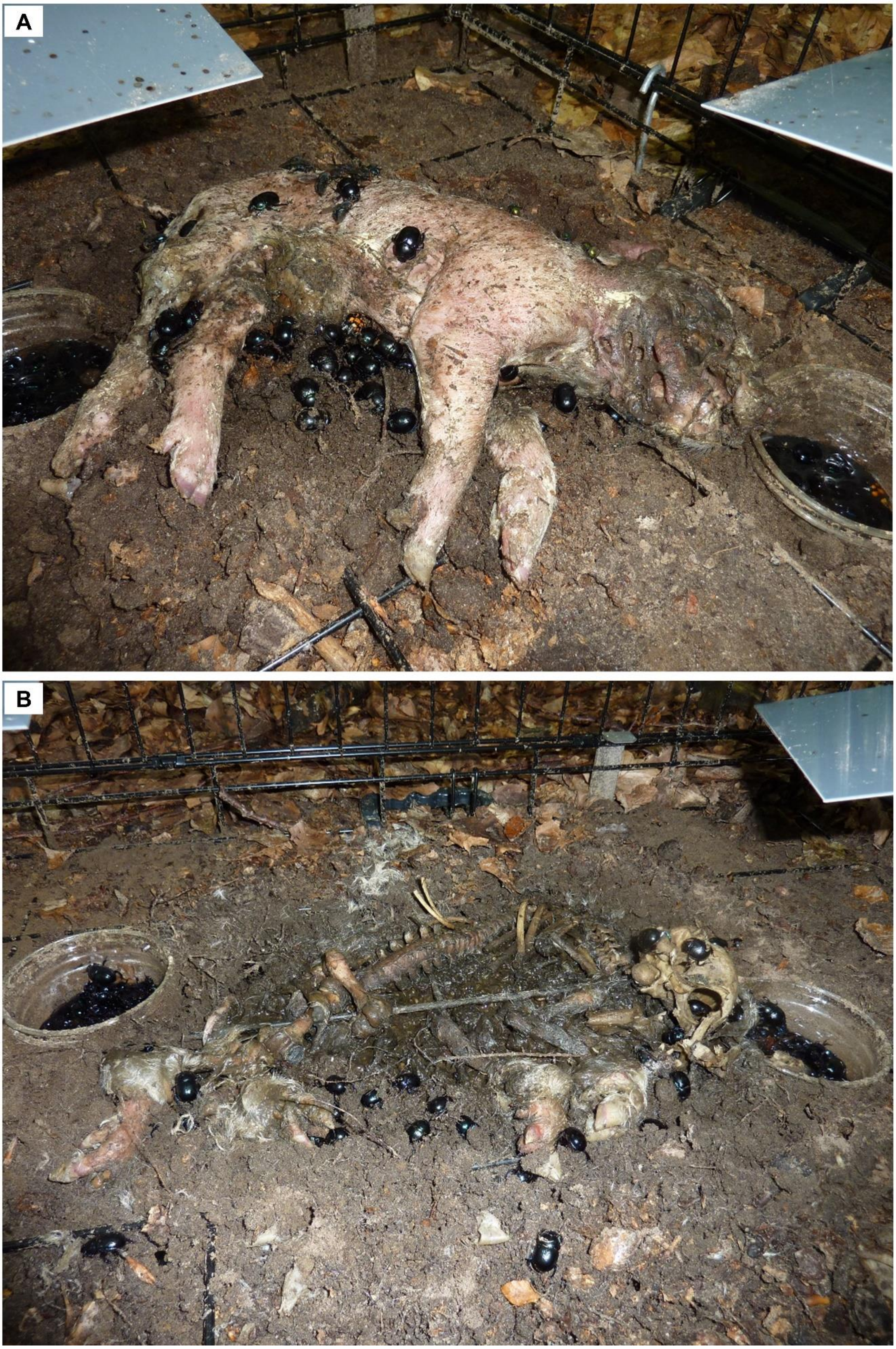

Figure S2. Mass assemblages of *Anoplotrupes stercorosus* at two piglet cadavers in (**A**) post-bloating and (**B**) advanced decay stage (forests in Schorfheide-Chorin region). Beetles underwent feeding on cadaveric fluids and almost became immobilized in the wet soil.

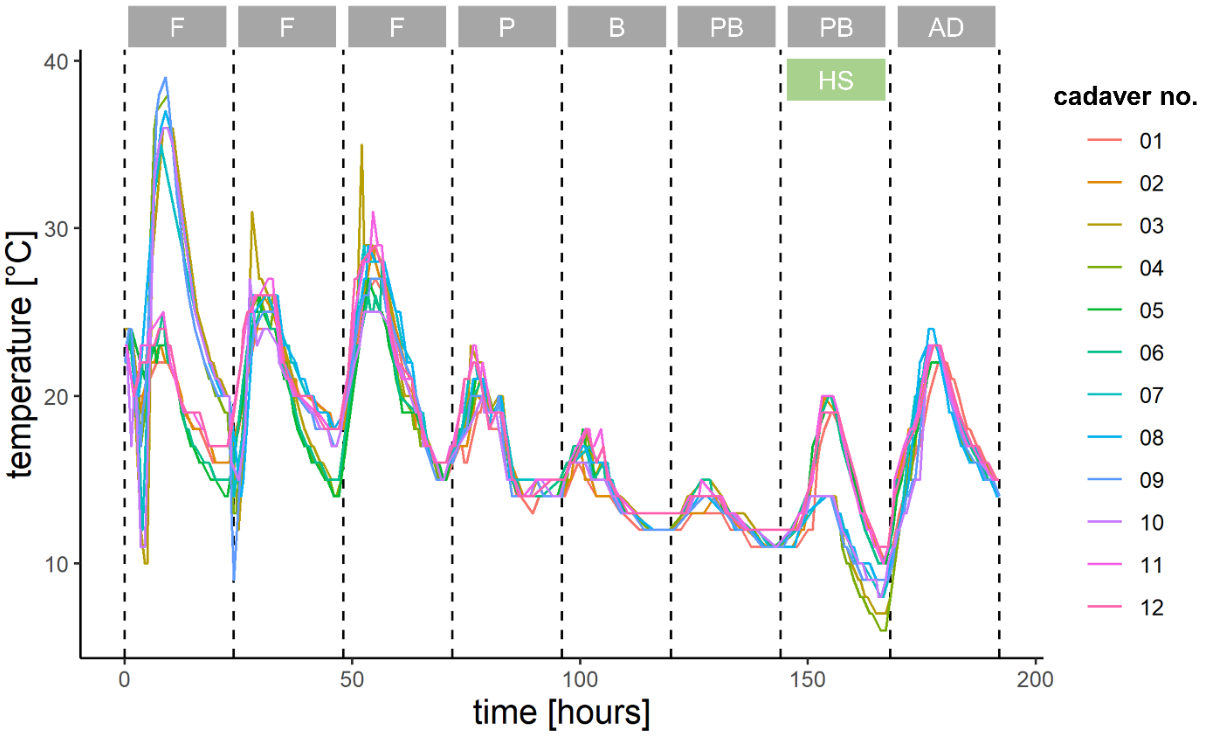

Figure S3. Temperature profile of the surroundings of 12 exposed piglet cadavers (01 – 12) over seven days. HS = Headspace sampling on day 6 after exposition. Decomposition stages: F = fresh, P = putrefaction, B = bloated, PB = post-bloating, AD = advanced decay.

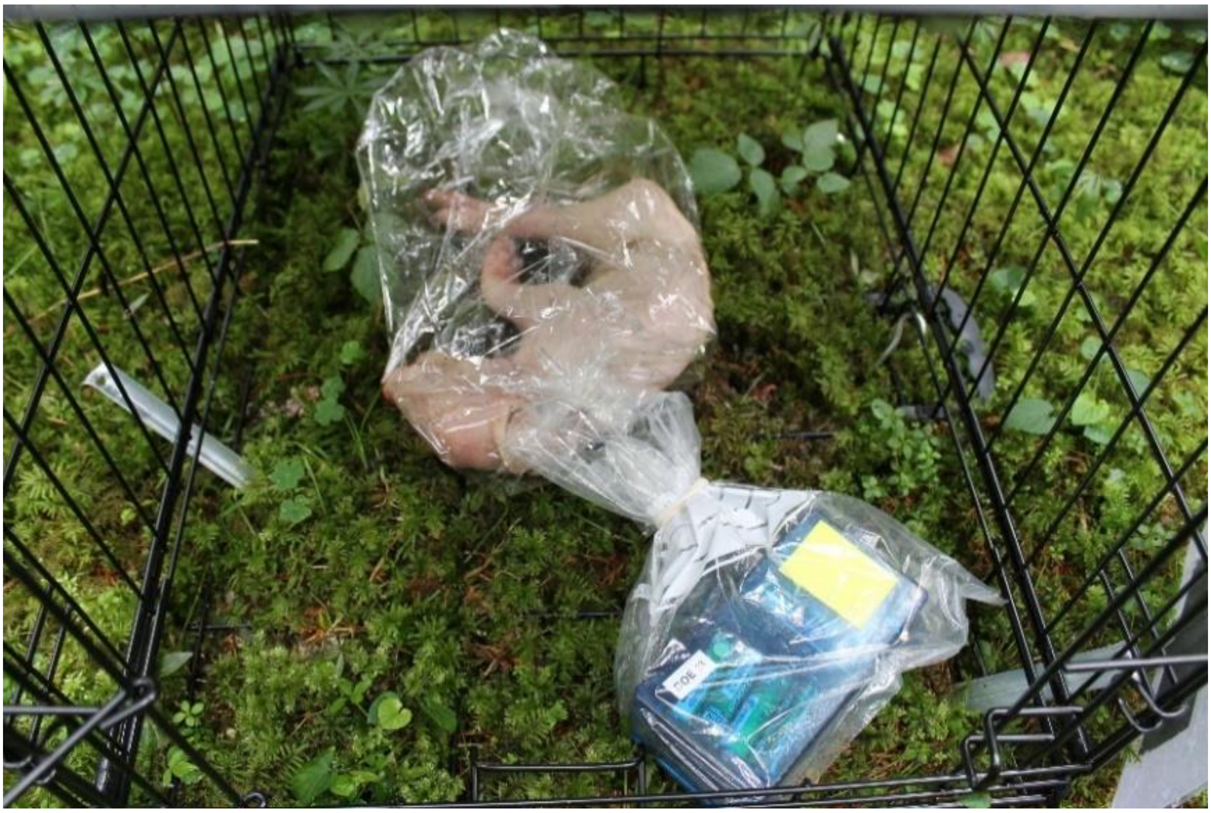

Figure S4. Sampling setup of dynamic headspace for cadaveric volatile organic compounds (VOCs) of a fresh piglet cadaver. In our experiment, we collected cadaver odor bouquets from piglets in post-bloating decay.

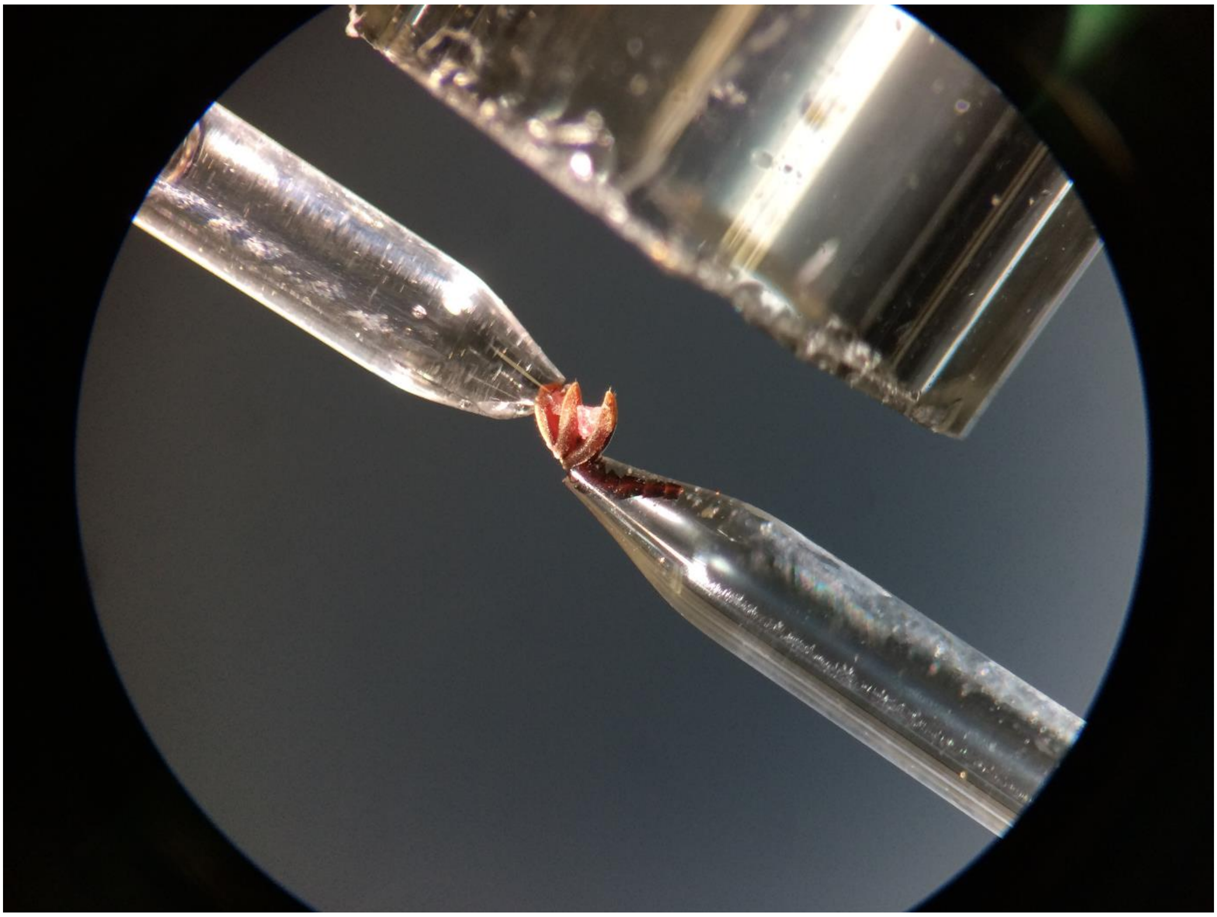

Figure S5. A lamellate antenna of the dung beetle *Anoplotrupes stercorosus* was fixed between two capillaries of the electroantennographic device. Two small dental wax pieces kept the lamella open for the incoming stimulus (cadaveric odor stream) towards the receptors.

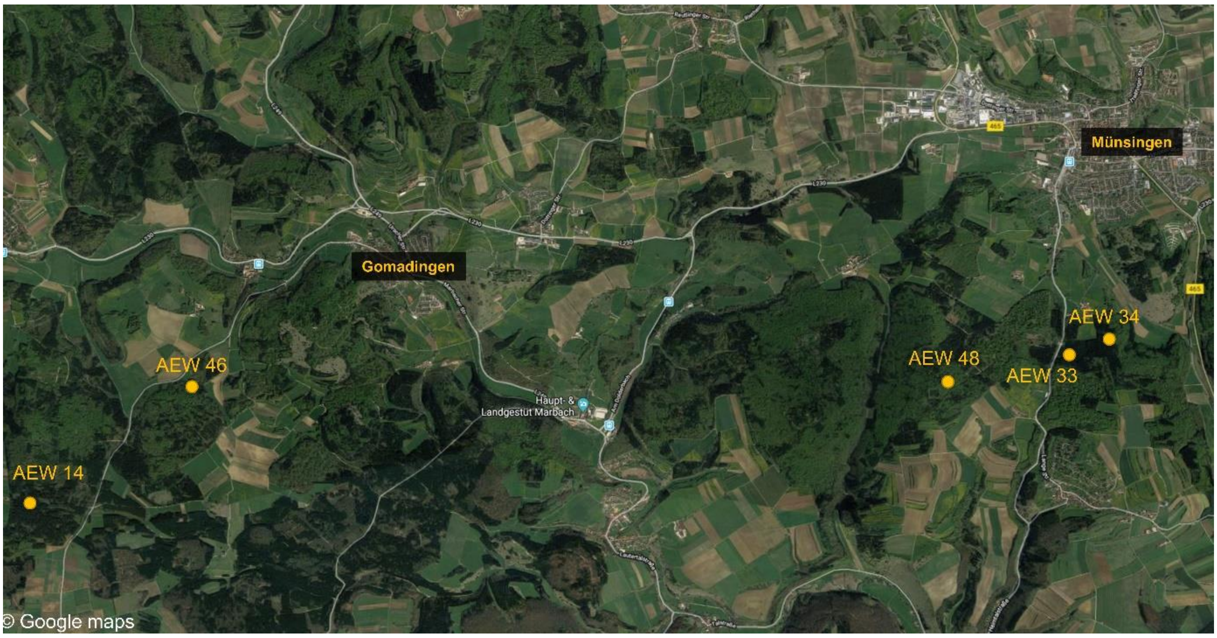

Figure S6. Location of the five forest plots AEW (Alb Exploratory Wald (*engl.* Forest) 14, 33, 34, 46, and 48 in the Schwäbische Alb near Gomadingen (GPS: 48° 23' 57.524'' N 9° 23' 28.306'' E) and Münsingen (GPS: 48° 24' 41.205'' N 9° 29' 52.929'' E) where we conducted our field assays. .

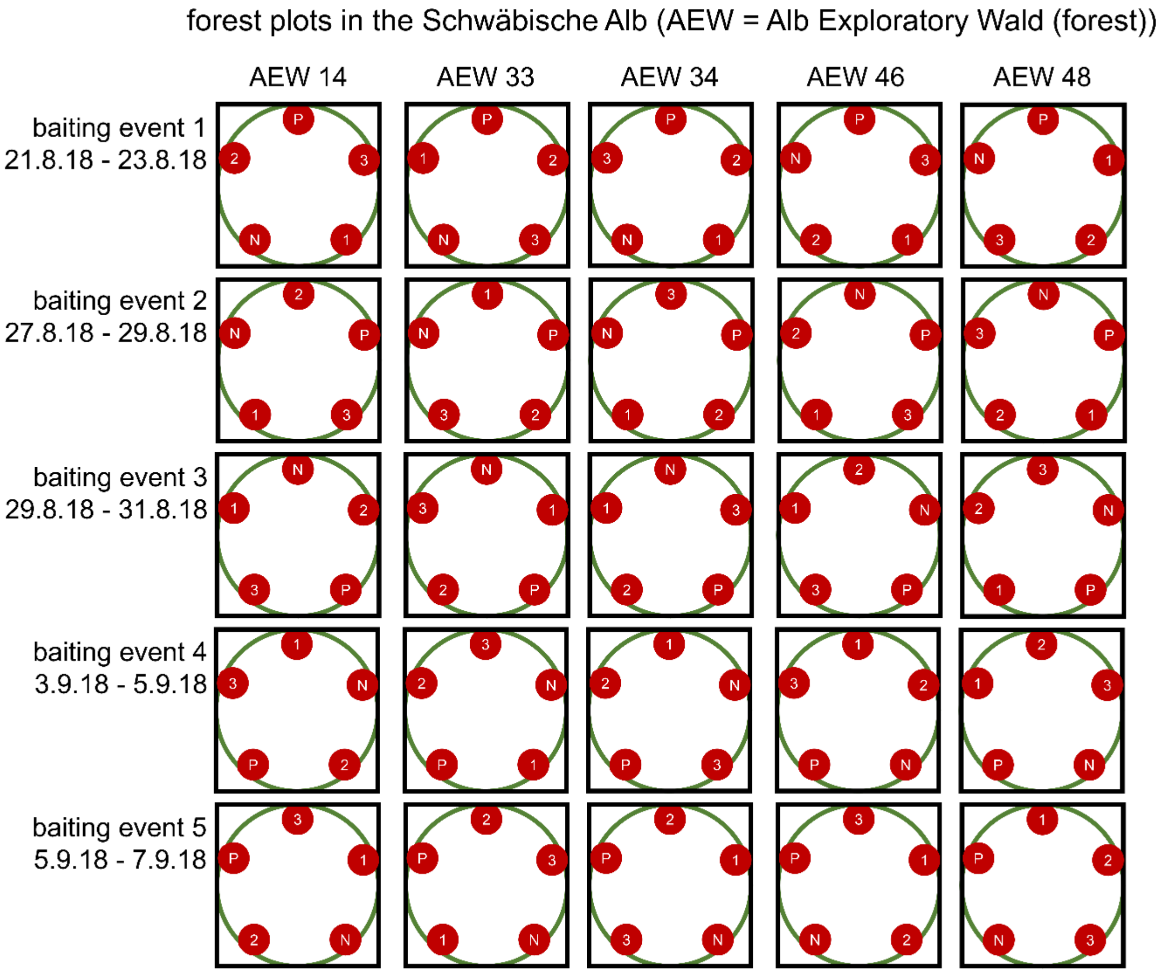

Figure S7. Baiting procedure across space (left to right: all five forest plots AEW 14, 33, 34, 36, 38) and time (five baiting events 1 - 5). Treatments 1, 2, 3, N, and P were rotated clockwise after each baiting event to avoid location effects. We paid special attention to ensure that, between each plot, treatments were arranged next to treatments deviating in treatment number to prevent cross-interactions. Treatment description: treatment 1 - complete mixture of all six EAD active compounds (benzaldehyde, dimethyl trisulfide, 3-octanone, 6-methyl-5-hepten-2-ol, nonanal, and dodecane), treatment 2 - three EAD active compounds (benzaldehyde, dimethyl trisulfide, and 3-octanone), treatment 3 - three EAD active compounds (6-methyl-5-hepten-2-ol, nonanal, and dodecane), treatment N - empty tube as negative control, treatment P - cadaver tissue in post-bloating stage as positive control.

Table S1. GC-EAD active compounds from the headspace sample pool used for the field assays. In the table we show their original total amounts (µg), their calculated pipette scheme to a sum of 250 µl, and adjusted pipette scheme (µl), together with the proportional pipette schemes for treatments 1 – 3.

**EAD active compounds**	amount from headspace sample (µg)	calculated original pipette scheme (µl)	adjusted final pipette scheme (µl)	**treatment 1** amount for 1 trap (µl)	**treatment 2** amount for 1 trap (µl)	**treatment 3** amount for 1 trap (µl)
dimethyl trisulfide	1.047	131	70	70	70	-
3-octanone	0.512	64	35	35	35	-
nonanal	0.193	24.1	98	98	-	98
6-methyl-5-hepten-2-ol	0.166	20.8	28	28	-	28
benzaldehyde	0.063	7.8	7	7	7	-
dodecane	0.021	2.6	7	7	-	7
**Sum**	2.001	~ 250	245	245	112	133

Treatment 1 - complete mixture: all six EAD active compounds (benzaldehyde, dimethyl trisulfide, 3-octanone, 6-methyl-5-hepten-2-ol, nonanal, and dodecane), treatment 2 - three EAD active compounds (benzaldehyde, dimethyl trisulfide, and 3-octanone), treatment 3 - three EAD active compounds (6-methyl-5-hepten-2-ol, nonanal, and dodecane).

Results

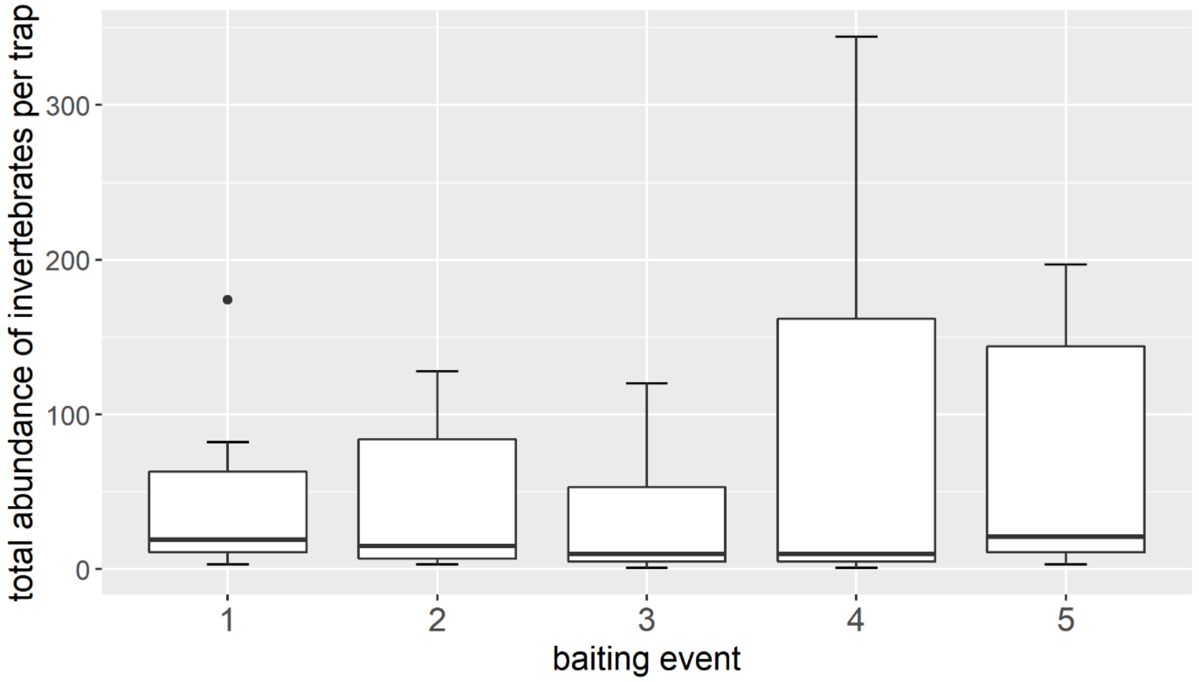

Figure S8. No significant difference of the total catch rate (total abundance of all lured invertebrates per trap) was observed among all baiting events (1 – 5) (Kruskal-Wallis test: $\chi^2 = 6.098$, df = 4, p = 0.192). Each box shows the median, 25 % percentile, 75 % percentile, and highest and smallest non-extreme value within a category.

Table S2. Abundance of insect and invertebrate taxonomic groups that were attracted by different treatments (1, 2, 3, N, and P).

**Taxonomic group**	**Abundance of attracted individuals**				
(total abundance)	treatment 1	treatment 2	treatment 3	treatment N	treatment P
A. stercorosus (220)	74	91	14	7	34
Silphidae (63)	21	38	1	0	3
Staphylinidae (472)	194	232	13	12	21
Flies (4438)	1864	2511	15	11	37
Slugs (77)	27	44	0	0	6
Carabidae (477)	121	85	112	69	90
Spiders (332)	50	63	68	79	72
Wasps (18)	4	4	2	2	6
Ants (40)	30	0	2	1	7
Isopods (67)	14	10	24	14	5
**Grand total (6204)**	**2399**	**3078**	**251**	**195**	**281**

Treatment 1 - all six EAD active compounds (benzaldehyde, dimethyl trisulfide, 3-octanone, 6-methyl-5-hepten-2-ol, nonanal, and dodecane), treatment 2 - three EAD active compounds (benzaldehyde, dimethyl trisulfide, and 3-octanone), treatment 3 - three EAD active compounds (6-methyl-5-hepten-2-ol, nonanal, and dodecane), treatment N - empty tube: negative control, treatment P - cadaver tissue in post-bloating stage: positive control.

Table S3. Significant differences in various attracted insect and other invertebrate taxonomic groups among the different treatments (1, 2, 3, N, and P).

**Taxonomic group** (total abundance)	**Overall** (Kruskal-Wallis)	Post-hoc Nemenyi test: **multiple comparisons among treatment groups** (N = 25 for each treatment)									
		complete vs blend 2	complete vs blend 3	complete vs empty tube	complete vs cadaver tissue	blend 2 vs blend 3	blend 2 vs empty tube	blend 2 vs cadaver tissue	blend 3 vs empty tube	blend 3 vs cadaver tissue	cadaver tissue vs empty tube
A. stercorosus (220)	H = 31.08 ***	ns	ns	**	ns	**	***	ns	ns	ns	ns
Silphidae (63)	H = 49.56 ***	ns	*	*	*	***	***	**	ns	ns	ns
Staphylinidae (472)	H = 58.92 ***	ns	***	***	***	***	***	***	ns	ns	ns
Flies (4438)	H = 94.71 ***	ns	***	***	***	***	***	***	ns	ns	ns
Slugs (77)	H = 37.72 ***	ns	*	*	ns	**	**	ns	ns	ns	ns
Carabidae (477)	H = 4.05	ns	ns	ns	ns	ns	ns	ns	ns	ns	ns
Spiders (332)	H = 4.90	ns	ns	ns	ns	ns	ns	ns	ns	ns	ns
Wasps (18)	H = 4.88	ns	ns	ns	ns	ns	ns	ns	ns	ns	ns
Ants (40)	H = 2.39	ns	ns	ns	ns	ns	ns	ns	ns	ns	ns
Isopods (67)	H = 1.67	ns	ns	ns	ns	ns	ns	ns	ns	ns	ns

Complete - all six EAD active compounds (benzaldehyde, dimethyl trisulfide, 3-octanone, 6-methyl-5-hepten-2-ol, nonanal, and dodecane), blend 2 - three EAD active compounds (benzaldehyde, dimethyl trisulfide, and 3-octanone), blend 3 - three EAD active compounds (6-methyl-5-hepten-2-ol, nonanal, and dodecane), empty tube: negative control, cadaver tissue: positive control. Significance levels: ns ($p > 0.05$), *($p \leq 0.05$), **($p \leq 0.01$),***($p \leq 0.001$).
